# A Method to Quantify Mouse Coat-Color Proportions

**DOI:** 10.1371/journal.pone.0005414

**Published:** 2009-04-30

**Authors:** Songthip Ounpraseuth, Tonya M. Rafferty, Rachel E. McDonald-Phillips, Whitney M. Gammill, Eric R. Siegel, Kristin L. Wheeler, Erik A. Nilsson, Craig A. Cooney

**Affiliations:** 1 Department of Biostatistics, The University of Arkansas for Medical Sciences, Little Rock, Arkansas, United States of America; 2 Department of Biochemistry and Molecular Biology, The University of Arkansas for Medical Sciences, Little Rock, Arkansas, United States of America; 3 Central Arkansas Veterans Healthcare Systems, Little Rock, Arkansas, United States of America; University Mainz, Germany

## Abstract

Coat-color proportions and patterns in mice are used as assays for many processes such as transgene expression, chimerism, and epigenetics. In many studies, coat-color readouts are estimated from subjective scoring of individual mice. Here we show a method by which mouse coat color is quantified as the proportion of coat shown in one or more digital images. We use the yellow-agouti mouse model of epigenetic variegation to demonstrate this method. We apply this method to live mice using a conventional digital camera for data collection. We use a raster graphics editing program to convert agouti regions of the coat to a standard, uniform, brown color and the yellow regions of the coat to a standard, uniform, yellow color. We use a second program to quantify the proportions of these standard colors. This method provides quantification that relates directly to the visual appearance of the live animal. It also provides an objective analysis with a traceable record, and it should allow for precise comparisons of mouse coats and mouse cohorts within and between studies.

## Introduction

Animals that exhibit coat-color variation and mosaicism have been used to evaluate a variety of effects in numerous studies [1]–[9]. In the yellow-agouti mouse model [10], the agouti protein antagonizes the melanocortin-1 receptor to promote the expression of the yellow pigment pheomelanin giving hairs a yellow color [11]. The *A^vy^* allele of *agouti* contains an intracisternal A particle (IAP) element inserted upstream of the *agouti* coding exons [12]. Over expression of agouti from this allele causes yellow hair. The *A^vy^* allele is epigenetically regulated and imprinted leading to only partial penetrance of the allele and to coats on most *A^vy^*/*a* mice that are a combination of yellow and agouti areas (mottling) [2], [10], [12]–[17].

Epigenetically determined coat color in this model is influenced by prenatal environmental exposure to compounds such as folate, vitamin B_12_ and methyl donors [2], [15], [16], [18], isoflavone phytoestrogens [19], DNA methyltransferase gene dosage [5] and maternal epigenetics [2], [10], [14]. To measure the influence of treatment factors on coat color, each mouse is classified into a pre-defined category or “phenotype” that constitutes one of several ordinal categories spanning the range of coat colors from “pseudoagouti” (no yellow) to “clear yellow” (no agouti spots). Depending on the laboratory, the number of ordinal categories can be two [10], three [14], four [5], five [16], [18], [19], or six [2], [15]. Although this ordinal-category framework is useful for assaying coat-color differences between treatment groups, the wide variation in the number of ordinal categories among laboratories makes it difficult to compare results from different studies. Even when two laboratories use the same number of ordinal categories, category definitions may differ (compare, for example, Cropley et al. [18] with Dolinoy et al. [19]), thus adding to the difficulty of comparison between studies. Laboratories employing fewer categories have the advantage of fewer definitions, but incur the costs of coarser calibration and attendant lower resolution. Finally, assigning a mouse to a coat-color category requires the grader to estimate the amount of yellow in the mouse's coat; this is currently a largely subjective process.

Here we describe a method to quantify the proportions of yellow and agouti in mouse coats. This method gives a numerical proportion and achieves approximately decile resolution over the population. The data generated by this method are continuous, not discrete, and thus will allow for meaningful use of regression, repeated-measures analysis, and other statistical procedures that go beyond the contingency-table methods currently employed. Most importantly, quantification by this method does not depend appreciably or systematically on the person assessing the mouse, and in that sense is objective. This method thus has the potential to allow for comparisons of experimental data between different studies and research groups.

## Materials and Methods

### Mice

Mice of the VY strain and heterozygous *A^vy^*/*a* genotype at the *agouti* locus were used [2], [15]. This VY strain (originally VY/WffC3Hf/Nctr-*A^vy^*) was a gift from Dr. George Wolff (National Center for Toxicological Research, Jefferson AR). This colony has been maintained at UAMS for seven years. Most such mice have yellow and agouti mottled coats. Mice were chosen with a range of levels of agouti in their coats to demonstrate the method, although we mainly chose mice between 0 and 50% agouti because, except for the colors used, this coloring process would be equivalent to coloring mice that are 50 to 100% agouti. Coats of these mice vary on the backs, sides, and heads, but not the abdomens. All animal experiments were approved by and conducted in accordance with the Institutional Animal Care and Use Committee of UAMS.

### Photography

Mice were photographed with a Nikon Coolpix 5000 digital camera under diffuse illumination from an Elmo model EV-368 illuminator. All images used the same illuminator, camera, camera settings, light blue background (Staples 110lb blue cardstock item number 490891) and were taken by the same photographer (TMR). Each mouse was photographed from the top (T) and from each side (left and right, L and R). Side views were between 30 and 60 degrees from vertical. Sets of photographs were taken on each of three days. Photographs of any one mouse were taken within one week, so that variations in coat appearance due to factors such as weight gain would be minimal. Photographs were taken of twelve mice. Images were saved as jpg files.

### Quantification of Mouse Coat Color

A copy of each mouse image was opened in a graphics program, the GNU Image Manipulation Program v.2.2 (GNU Project, http://www.gnu.org/), which is a raster graphics editor. On these pictures, background regions were selected using the Lasso tool (with Antialiasing inactive) and colored red with the Paint Bucket tool. The background, eyes, ears, paws, tail, bald spots and any areas that were not mouse coat were colored red. Agouti regions were selected with the Magic Wand tool, and the threshold was increased or decreased until the selected areas most closely matched the regions of agouti coat. The selected agouti regions were then colored brown with the Paint Bucket tool. The Select Regions By Color tool was then used to select the remainder of the coat (yellow) and color it yellow with the Paint Bucket tool. The Red, Green, Blue (RGB) values used for each color are given in Table 1. The Flatten Image command was used and the new file was saved. Each mouse image was colored at least two times and colorings were matched for correspondence of natural agouti and yellow regions of the mouse image with the agouti and yellow regions of the coloring. Two individuals called raters matched colorings with images. The two raters acted independently of each other, and their results were subsequently evaluated by a third individual for quality-assurance purposes. The brown and yellow proportions were calculated using a program written in the R language (http://cran.r-project.org/) [20]. The following R script computes the brown and yellow proportions for each mouse.

**Table 1:** RGB proportions for the red, brown, and yellow colors.

Color	R	G	B
Red	254	0	0
Brown	96	57	18
Yellow	255	255	0

library(“rimage”)

library(base)

x<-read.jpeg(“filename.jpg”)

plot(x)

x1<-as.vector(x[,,1])

x2<-as.vector(x[,,2])

x3<-as.vector(x[,,3])

y<-cbind(x1,x2,x3)

counts<-ifelse(y[,2]< = .05,1,ifelse(y[,2]>.05 & y[,2]< = .3,2,3))

table(counts)

z<-as.matrix(table(counts))

pct<-z[2∶3,]

colors<-c(“brown”,“yellow”)

color_pct<-round(pct/sum(pct)*100,1)

color_pct<-paste(color_pct, “%”, sep = “”)

pie(pct,main = “Mouse Coat Color Proportions”, col = colors, label = color_pct, cex = .8)

legend(1.5,.5,c(“Brown”,“Yellow”),cex = .8,fill = colors)

In the above script the filename.jpg should be replaced with the actual name of the file (e.g. Mouse5colored.jpg) to be analyzed.

### Data Analysis

For each of the three days of photographs and for each rater, the mouse's left-side and right-side yellow proportions were averaged together and called “Sides”, while its Top yellow proportion was kept separate. Twelve measurements per mouse (six Top and six Sides) were thus obtained, and the difference between Top and Sides was calculated. Additionally, the Top measurements, and likewise the Sides measurements, were averaged within each mouse across raters, across three days of photographs, and both, and the differences between Top and Sides averages were calculated, in order to study the effect of aggregating a mouse's measurements from different times and raters. Top, Sides, and difference were summarized by rater, time, and average as means and standard deviations (SDs). The Two One-Sided Tests (TOST) procedure [21] was used at 5% alpha to determine whether measurements from Top and Sides were equivalent to within ±10 percentage points. Values from Rater 1 versus Rater 2 were plotted via both standard scatterplot and Bland-Altman plot [22]; for the latter, 95% limits of agreement were calculated using the modification of Bland and Altman [23].

For the continuous mouse coat-color-proportion measurements derived from our proposed method to be useful as a research tool, they must have a high degree of repeatability. Variations in continuous measurements can arise from inconsistent measurement practices as well as differences in (or wearing out of) equipment. We quantified the within- and between-observer variations by re-measuring the same mouse on three different occasions by two different raters.

Because mice were rated by two individuals, measurements of the level of agreement between raters were needed. Many methods-development papers try to measure agreement by calculating the Pearson correlation coefficient between the raters or methods, but this is inadequate [22]. Van Belle [24] notes that the Pearson correlation coefficient captures only precision while ignoring both bias and differences in dynamic range. However, a different measure, the Intraclass Correlation Coefficient [25], incorporates the bias and dynamic-range differences into the calculation; as a result, this measure is widely accepted in the social sciences as the preferred measure of agreement between two raters or two methods.

The intraclass correlation coefficient (ICC) assesses the relative extent to which multiple continuous measurements taken by different individuals, or by the same person on different occasions, are related. In general, the ICC describes the ratio of two variances
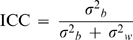
where σ*^2^_b_* denotes the variation between subjects (i.e., between mice), and σ^2^ *_w_* is the pooled variance within subjects. The range of the ICC may be between 0 and 1. A high ICC value would indicate that there is little variation in the coat-color percentages computed for each mouse by the raters.

Shrout and Fleiss [25] describe six ICC notations and their uses for reliability. For our study, we report the ICC(2,1), for which all mice are assessed by the same raters (judges) who are assumed to be a random subset of all possible judges. We used SAS® Version 9.1 (SAS Institute, Carey, NC) along with two user-written macros to calculate the ICCs and their 95% confidence intervals, INTRACC by Robert Hamer (http://www.psych.yorku.ca/lab/sas/intracc.htm), and ICC macro from Douglas Steinley and Phillip Wood (http://ourworld.compuserve.com/homepages/jsuebersax/icc.htm).

## Results

**Figure 1:**
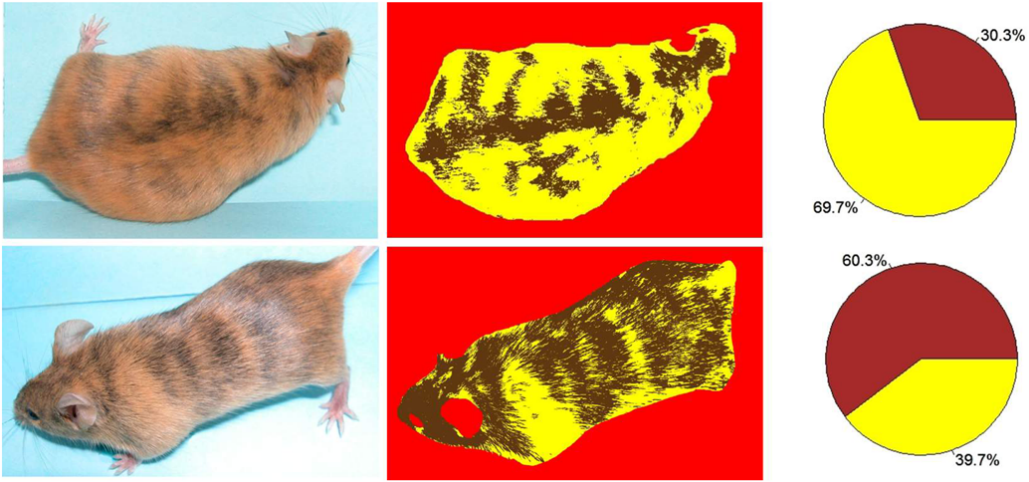
Photographs, colorings, and pie charts of mice. Photographs of mice were processed as described in the text to produce yellow areas of uniform color and agouti areas of uniform color that closely matched yellow and agouti areas on the live mouse. A script was used to generate a pie chart from these areas of uniform color to determine the percentage of yellow and agouti in the mouse. Two examples are shown here.

We used a group of mottled mice that varied from mainly yellow to mainly agouti to quantify their coat color patterns. Each mouse was photographed at three different angles (left, right and top) on each of three different days. Two individuals, called raters, colored copies of each photograph. In some cases raters had the option of choosing from two or more existing colorings of a photograph or producing their own coloring. Raters' matches of colored pictures were screened for accurate match by a third person. Except for this screening by a third person, raters were independent and did not communicate with each other about mouse coloring. We mainly colored mice between 0 and 50% agouti because, except for the colors used, this coloring process would be equivalent to coloring mice that are 50 to 100% agouti. Example photographs, colorings and quantifications are shown in Figure 1.

An important part of the method is matching the coat color pattern on each mouse to a standardized color version that can be readily quantified using a computer. Because this part of the method relies on visual matching, we had two raters color mice, and we then compared their results.

**Figure 2:**
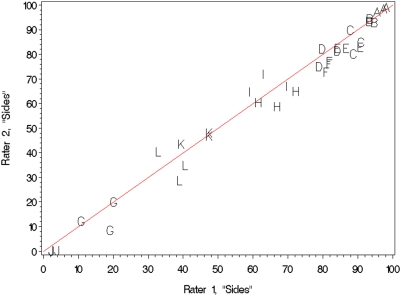
A plot of the percent yellow in the coats of mice evaluated from their sides. Two raters matched colorings on 12 mice on three occasions by examination of each mouse from its left and right sides. Vertical and horizontal axes denote the average percent yellow of the mouse's left and right sides. Letters denote the identity of the mouse being rated, while the red diagonal line denotes the line of equality between percentages assigned by the two raters.

**Figure 3:**
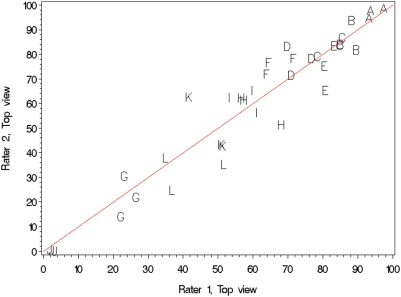
A plot of the percent yellow in the coats of mice evaluated from the top. Two raters matched colorings on 12 mice on three occasions by examination of each mouse from the top. Vertical and horizontal axes denote the percent yellow assigned by each rater. Letters denote the identity of the mouse being rated, while the red diagonal line denotes the line of equality between percentages assigned by the two raters.

**Figure 4:**
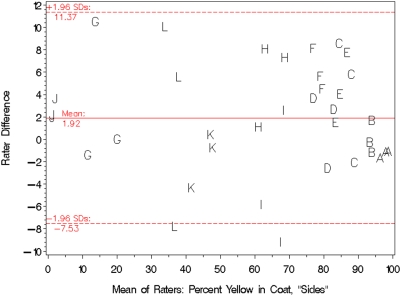
A Bland-Altman plot of the data shown in Figure 2. The horizontal and vertical axes respectively show the mean and difference in coat-color percentages assigned by the raters. Letters denote the identity of the mouse being rated. The solid line depicts the mean of the differences between raters, and the area between the dotted lines delineates the 95% limits of agreement.

**Figure 5:**
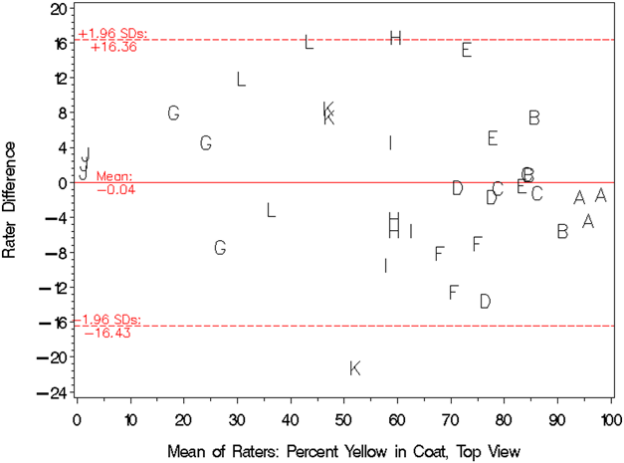
A Bland-Altman plot of the data shown in Figure 3. The horizontal and vertical axes respectively show the mean and difference in coat-color percentages assigned by the raters. Letters denote the identity of the mouse being rated. The solid line depicts the mean of the differences between raters, and the area between the dotted lines delineates the 95% limits of agreement.

Photographs of mice were taken from each side at a substantial angle to vertical (“Sides”) as well as along the vertical (“Top”). Figure 2 and Figure 3 show standard scatter plots of percents yellow in the Sides and Top, respectively, of each mouse as determined by rater 1 versus that determined by rater 2. The Pearson Correlation Coefficients between raters were 0.989 (*P*<0.0001) for Figure 2 and 0.961 (*P*<0.0001) for Figure 3. The diagonal line in both figures is “the line of equality on which all points would lie if the two [raters] gave exactly the same reading every time” [22]; distance from the line of equality indicates the amount by which the two raters' measurements disagree. A high-resolution view of rater disagreement is shown in the Bland-Altman plots of Figure 4 and Figure 5, for mouse Sides and Top, respectively, in which the difference between raters is plotted against the average between them. For percents yellow in the sides of the mice, the mean difference between raters was 1.92% and the 95% limits of agreement were −7.53% to +11.37%, a spread of around 19 percentage points. For percents yellow in the tops of the mice, the mean difference between raters was −0.04% and the 95% limits of agreement were −16.43% to +16.36%, a spread of almost 33 percentage points.

Table 2 shows means and SDs for Top, Sides, and their difference, by rater, time, and both, and after averaging Top and Sides over rater, time, and both. In all cases, the mean of coat color proportion determined from the top was somewhat less than from the sides, ranging from −0.23 to −5.48 percentage points among the different raters and times, and equal to −3.48 percentage points using Top and Sides averaged over both time and rater. However, for all rows of Table 2, the two one-sided 95% confidence limits (TOS CL) on the mean difference between Top and Sides measurements both lie inside equivalence-limit boundaries at +10 and −10 percentage points. This establishes via the TOST procedure that the Top and Sides measurements are equivalent to within ±10 percentage points.

**Table 2:** Means and SDs of coat percent yellow, as measured from the top and sides at different times by different raters.

Color Measurement Parameter^A^		Top view	Sides^B^	Difference^C^	
		Mean (SD)	Mean (SD)	Mean (SD)	TOS CL^D^
Time1	Rater1	61.1 (27.9)	63.4 (30.3)	2.38 (6.00)	−0.73–5.49
Time1	Rater2	59.7 (27.8)	62.7 (31.2)	3.00 (7.31)	−0.79–6.79
Time1	averaged	60.4 (27.5)	63.1 (30.7)	2.69 (5.55)	−0.19–5.57
Time2	Rater1	58.4 (27.3)	63.8 (30.6)	5.35 (6.27)	2.09–8.60
Time2	Rater2	60.4 (27.6)	60.6 (32.7)	0.23 (9.33)	−4.60–5.07
Time2	averaged	59.4 (27.2)	62.2 (31.5)	2.79 (6.33)	−0.49–6.07
Time3	Rater1	60.4 (27.8)	63.3 (33.3)	2.89 (8.45)	−1.49–7.27
Time3	Rater2	59.8 (32.8)	61.4 (31.2)	1.52 (6.68)	−1.95–4.98
Time3	averaged	60.1 (30.1)	62.3 (32.2)	2.20 (4.66)	−0.21–4.62
averaged	Rater1	59.9 (27.4)	63.5 (31.3)	3.54 (6.11)	0.37–6.71
averaged	Rater2	60.0 (29.0)	61.6 (31.5)	1.58 (4.60)	−0.80–3.97
averaged	averaged	60.0 (28.1)	62.5 (31.4)	2.56 (4.73)	0.11–5.01

A Coat colors were measured as percentage yellow by two different raters each at three specific times within a one-week period. Measurements were also averaged by rater, time, or both.

B Sides were calculated as the average of the measurements from the left side and right side.

C Difference was calculated as Sides–Top. For “averaged” measures, the difference was calculated between the averaged Top and Sides measures.

D Two One-Sided 95% Confidence Limits on the mean difference between Top and Sides. Under the Two One-Sided Tests (TOST) procedure, the Top and Sides are equivalent (at 5% alpha) to within ±10 percentage points if the two one-sided 95% confidence limits both lie inside equivalence-limit boundaries located at +10 and −10 percentage points.

Using coat-color measurements averaged across the three time points, the agreement between raters was high whether evaluated using photos taken from the sides (ICC = 0.994, Table 3) or from the top (ICC = 0.986, Table 3).

**Table 3:** Inter-rater reliability estimate between the two raters for time-averaged measurement derived from the indicated photographic angle.

Test	ICC^A^	95% CI for ICC
% Yellow (Sides^B^)	0.994	[0.986, 0.998]
% Yellow (Top^C^)	0.986	[0.971, 0.995]

A Intraclass Correlation Coefficient, type (2,1) in Shrout and Fleiss [25] notation.

B Sides: Average of two measurements per mouse, each derived from photos taken on the left and right sides of the mouse.

C Top: Single measurement per mouse, derived from photo taken from vertical (top) angle.

For the method to be reliable, photos of the same mouse should not differ greatly from each other, especially when taken from approximately the same angle. Quantification from colored images was compared for the three days for each rater. The agreement between different days was high for both raters whether evaluated using photos from the sides (ICC = 0.988 and 0.988, Table 4) or photos from the top (ICC = 0.953 and 0.974, Table 4).

**Table 4:** Intra-rater reliability of each rater measuring the percentage of yellow coat color in each mouse on three different days from the sides^A^ and from the top.

Angles and Raters	ICC^B^	95% CI for ICC
Sides, Rater 1	0.988	[0.975, 0.996]
Sides, Rater 2	0.988	[0.976, 0.996]
Top, Rater 1	0.974	[0.948, 0.991]
Top, Rater 2	0.953	[0.908, 0.983]

A Sides: Average measure from left- and right-side photos per mouse.

B Intraclass Correlation Coefficient, type (2,1) in Shrout and Fleiss [25] notation.

Quantification from colored images of the two raters was compared for the three days on which photographs were taken. The agreement between raters was high for pictures taken on different days, whether from the sides (ICC = 0.980 to 0.994, Table 5) or from the top (ICC = 0.956 to 0.966, Table 6).

**Table 5:** Inter-rater reliability between the two raters on the individual days, evaluated using the average of measurements from photos taken from the left and right sides.

Test	Measurement	N	ICC^A^	95% CI for ICC
% Yellow	Day 1	12	0.994	[0.988, 0.998]
	Day 2	12	0.980	[0.958, 0.993]
	Day 3	12	0.987	[0.956, 0.996]

A Intraclass Correlation Coefficient, type (2,1) in Shrout and Fleiss [25] notation.

**Table 6:** Inter-rater reliability between the two raters on the individual days, evaluated using measurements from photos taken from the top angle only.

Test	Measurement	N	ICC^A^	95% CI for ICC
% Yellow	Day 1	12	0.954	[0.905, 0.984]
	Day 2	12	0.966	[0.929, 0.988]
	Day 3	12	0.956	[0.910, 0.985]

A Intraclass Correlation Coefficient, type (2,1) in Shrout and Fleiss [25] notation.

## Discussion

We describe a method to quantify the coat color percentage in live yellow-agouti mottled mice using digital photographs saved as jpg files. This method uses available, free, established software, the GNU Image Manipulation Program, and a short script written in the R programming language.

Photographs of the same mouse taken on different days within a one-week period yielded very similar results from one photo session to the next when we took reasonable steps (e.g., using the same diffuse light source, same camera with the same settings, same background material and the same photographer) to insure consistency of conditions among sessions. In general, we found little variation among quantifications of pictures taken on different days, indicating that multiple photographs provide little advantage for quantifying coat colors. Two different raters matched colorings to mice, and obtained, on average, very similar coat-color percentages. Each coloring was evaluated by a third individual who screened colorings for a reasonable match. In a few cases (less than 10% of colorings), raters were asked to recolor images. Fayers and Machin [26] recommended that the ICC should exceed 0.90 for techniques that are to be used to assess individuals in clinical practice. Their recommendation clearly applies to techniques for assessing individual mice in laboratory research. Not only did all our ICCs exceed the 0.90 threshold, but all the lower 95% confidence limits on our ICCs exceeded it, thus establishing that our technique has the inter-rater reliability required by Fayers and Machin [26].

Our results indicate that measuring the coat color of mice from only top-view photographs should be sufficient for most purposes. The proportions yellow obtained from top-view photos averaged about 3.5 percentage points lower than those obtained from side-view photographs. This difference is small compared to the time saved from taking and coloring fewer pictures when only top views are used. Application of a well-established statistical equivalence-testing procedure [21] to the difference between top-view and side-view measurements demonstrated that the two were significantly equivalent to within ±10 percentage points for all combinations of raters, times, and averagings.

Transgenic, knockout and other genetically modified mice are typically made using embryonic stem cells, which are then implanted in an early embryo [7], [8]. Highly chimeric mice are used as an indicator of those most likely to carry a germ-line with the desired modification [7], [8], [27]–[29]. Similar embryonic stem cell methods have been developed for rats [9], [30]. The method we describe here could provide a quantitative means to select mice and rats with the highest levels of coat color chimerism. In turn, these animals may have the highest levels of germ line chimerism.

Coat color assays provide patterns of epigenetic variation over a wide area of tissue (skin and hair). Further, DNA and other molecular components can be extracted from plucked hairs (including follicles) using hair color as a guide (our unpublished results). Coat color models of epigenetics usually assay the epigenetics of one particular genetic locus and of one tissue (skin). Coat color methods alone do not address the molecular basis of epigenetic regulation. In contrast, molecular methods such as DNA methylation analysis [14]–[16], [31]–[35] or chromatin immunoprecipitation [36], [37] can be applied to many loci and tissues. In addition to skin, large patterns of epigenetic mosaicism probably exist in many other tissues (e.g. liver, brain). Except for patterns that can be detected visually or by immunohistochemistry [33], analyses of epigenetic differences in tissue sections at high spatial resolution is likely to be very labor intensive.

Quantification of color in skins removed from mice has been reported [38]. The method used proprietary software to analyze mouse skins after sacrifice but offered few other details. Although the method was used to determine percentage mottling, the vast majority of mice were less than 50% “black” and the highest percent mottling reported in a large mouse population was about 80% “black”. It is unclear how data from this method would relate to live mice or to the visual appearance of mice, many of which are 80 to 100% agouti. Our method provides quantification that relates directly to the visual appearance of the live animal.

Our method has two main advantages over the widely used ordinal-category methods for live mice. First, it is quantitative and provides data that can be analyzed and compared with other measures with greater precision than ordinal-category methods. Second, it provides a record of the mouse image and how the image was colored. This record can be reevaluated as needed.

We use the yellow agouti mouse model of epigenetic variegation to demonstrate this method. However, it should be useful for many types of studies where coat-color mosaicism or coat-color phenotype is important. This method by which the proportion of coat color on a live mouse can be objectively quantified should allow for precise comparisons of mouse coats within and between studies.
